# Alcohol extract from *Vernonia anthelmintica willd (L.)* seed counteracts stress-induced murine hair follicle growth inhibition

**DOI:** 10.1186/s12906-019-2744-9

**Published:** 2019-12-17

**Authors:** Qian Wang, Yongxin Wang, Silin Pang, Jia Zhou, Jie Cai, Jing Shang

**Affiliations:** 1 State Key Laboratory of Natural Medicines, China Pharmaceutical University, Nanjing, 210009 JiangSu Province People’s Republic of China; 2 Jiangsu Key Laboratory of TCM Evaluation and Translational Research, China Pharmaceutical University, Nanjing, 210009 People’s Republic of China; 3 The Pharmaceutical Department, Nanjing Zhong-da Hospital, School of Medicine, Southeast University, Nanjing, 210009 People’s Republic of China; 4 Qinghai Key Laboratory of Tibetan Medicine Pharmacology and Safety Evaluation, Northwest Institute of Plateau Biology, Chinese Academy of Sciences, Xining, 810008 People’s Republic of China

**Keywords:** *Vernonia anthelmintica (L.) willd* extract (AVE), Hair follicle, Chronic restraint stress, Melanogenesis, Nerve fibers, Substance P

## Abstract

**Background:**

*Vernonia anthelmintica (L.) willd* is a traditional urgur herb in China for a long history. Its alcohol extract (AVE) has been proved to promote hair follicle growth in C57BL/6 mice. We conducted this study to investigate the hair-growth effects of AVE in stressed mice and its possible mechanism of action.

**Methods:**

The hair-follicle growth effects of AVE were examined by in vivo and in vitro study. We exposed C57BL/6 male mice to chronic restraint stress to induce murine hair follicle growth inhibition. The effects of AVE were examined by histological analysis, immunofluorescence for Ki67 and cytokeratin 19 immunoreactivity, western blot assay in tyrosinase and related proteins expressions and immunofluorescence for nerve fibers. In organ culture of mouse vibrissae follicles, we used substance P as a catagen-inducing factor of hair follicle growth, and measured the elongation of hair shafts and expression of neurokinin-1 receptor protein by application of AVE.

**Results:**

Our results showed that AVE counteract murine hair follicle growth inhibition caused by chronic restraint stress via inducing the conversion of telogen to anagen and inhibiting catagen premature, increasing bulb keratinocytes and bulge stem cells proliferation, promoting melanogenesis, and reducing the numbers of substance P and calcitonin gene-related peptide nerve fibers. Furthermore, AVE also counteracted murine hair follicle growth inhibition caused by substance P in organ culture.

**Conclusion:**

These results suggest that AVE counteract stress-induced hair follicle growth inhibition in C57BL/6 mice in vivo and in vitro, and may be an effective new candidate for treatment of stress-induced hair loss.

## Background

As one of the most common skin diseases, hair loss has negative social impact on patients by reducing their quality of life although it is not life-threatening [1–3]. Clinical experience has long suggested that psychological stress plays an important role in triggering and exacerbating hair growth disorders [4]. As the references reported, alopecia areata (AA), telogen effluvium, lichen planopilaris (LPP) may have relations with stressful events [3, 5, 6].

As the largest organ of human body, skin protect human from exogenous stressors [7]. And hair follicle is an important skin appendage, which displays a hair cycle with three periods of active growth (anagen), degenerative (catagen), and relative resting (telogen) [8, 9]. In all mammalian species, follicular melanogenesis is coupled to anagen, ceases during catagen, absent during telogen. Other from human, melanocytes of C57BL/6 mice only locate in the hair bulb region, the dorsal skin color appears to be pink in telogen and changes to be black in the anagen stage [10]. Therefore, hair follicles of C57BL/6 mice appears ideally suited to study hair follicle and screen hair growth promoting agents.

Stress might act as a precipitating factor in the onset or exacerbation of hair loss through psychosomatic mechanism [11, 12]. Previous research have identified that psychological stress can alter the hair cycle and affect hair growth via increasing apoptotic cells, inhibiting hair bulge stem cells and hair bulb keratinocytes proliferation, promoting mast cell degranulation, inducing premature catagen and neurogenic inflammation [13]. Sensory and autonomic nerve fibers are richly innervated with hair follicles. There is an intimate interaction between the cutaneous innervation and the progression of hair follicle cycling. Mountains of evidences have shown that hair growth is profoundly influenced and regulated by neuropeptides involving in systemic stress responses [14]. Substance P (SP) and calcitonin gene-related peptide (CGRP) have been confirmed to effectively manipulate skin and immune cell functions, including cell proliferation, antigen presentation, and cytokine production under both normal and pathological conditions in C57B/6 mice model [15, 16]. Furthermore, in the absence of a functional perifollicular innervation, both two neuropeptides have similar inhibited effects on organ-cultured hair follicle growth [16]. Arck et al. found that the percentage of SP and CGRP sensory neurons may be up-regulated under stress exposure in murine dorsal skin [17]. The neurobiological, neurorendocrine, and neuroimmunological changes associated with psychoemotional stress provide us a novel perspective and therapeutic option to study hair growth problems.

At present, FDA only approves two synthetic agents, minoxidil and finasteride, for the treatment of hair loss [18]. Minoxidil is used to treat alopecia areata, chemotherapy-induced alopecia, hair transplant, and finasteride is efficacious for androgenetic alopecia [19, 20]. However, both of them have undesirable side effects and there was no literature had been reported that these two agents had psychosomatic mechanism [19, 21]. Therefore, it is urgent to explore more alternative agents with a limited side effect particularly natural products.

*Vernonia anthelmintica (L.) willd* grow in minority regions of India and Pakistan [22]. It is reported that its fruit extract (AVE) can promote hair follicle growth in C57BL/6 mice [23]. Based on our previous studies, we found that AVE may affect mental status in C57BL/6 mice. However, whether AVE could have effects on stress-induced hair follicle growth remain unknown. In this study, we focused on the effects of AVE on stress-induced hair follicle growth inhibition both in vitro and in vitro. Furthermore, we investigated the possible mechanism on hair follicle growth by application of AVE.

## Materials and methods

### Preparation of AVE

The seeds of *Vernonia anthelmintica (L.) willd* were collected from Aksu region in Xinjiang, China. Botanical samples were authenticated by Professor Tiemin Ai (College of Pharmacy, Peking University Health Science Center). Its voucher specimen has been deposited at herbarium of School of Traditional Chinese Medicine, China Pharmaceutical University (voucher No. 2011–1105).

We prepared the extract through solvent extraction by 60% ethanol according to the previous study [24]. The dark brown powder of AVE was obtained after being degreased and dried, the components have been identified in the HPLC profile [24].

### Animals

Syngenic, male C57BL/6 mice (5~6 weeks old, weighing 18-22 g) were obtained from Comparative Medicine Centre of Yangzhou University (China). All mice were left undisturbed and acclimatized for 7 days prior to the onset of studies at Experimental Animal Center of Southeast University (China). The standard conditions of animal facility were maintained as following: temperature 21–24 °C; 12 h light/dark cycle (lights on 06:00–18:00); humidity 50–60%. Sterilized water and food were provided *al libitum* during this period. All experimental procedures and animal care were approved by the Animal Study Committee of China Pharmaceutical University (Permit Number: PMY33069N).

### Stress application

**Fig. 1:**
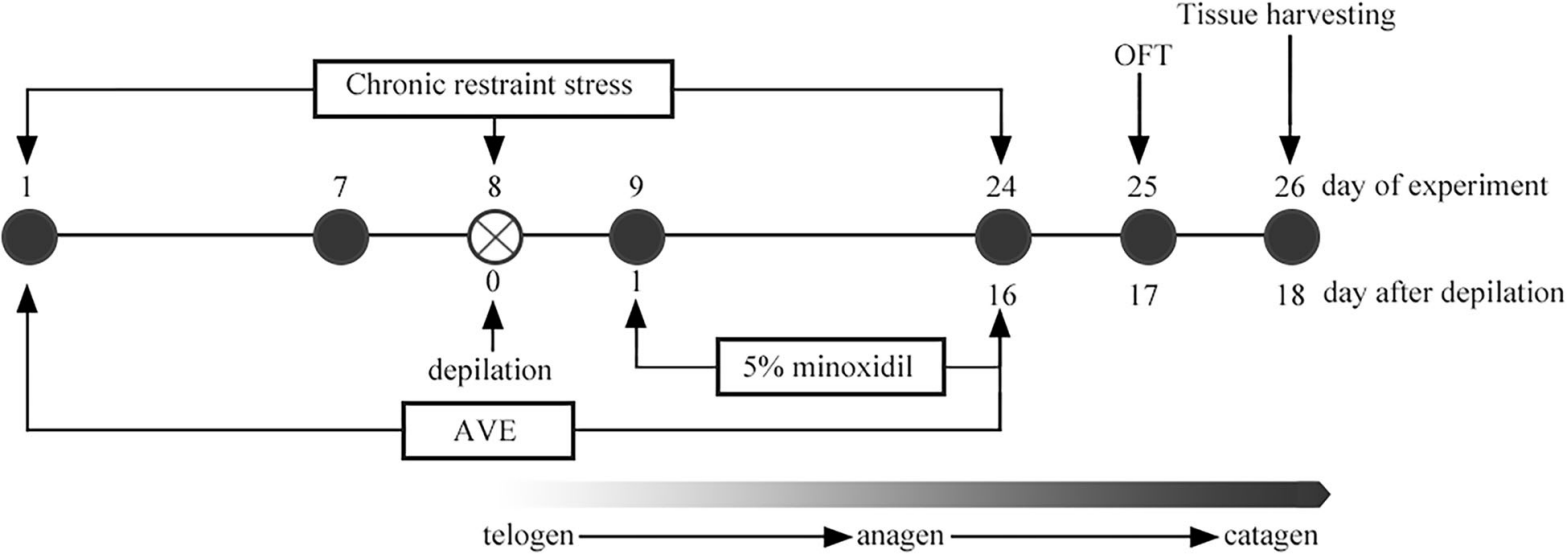
Time schedule of the experiment. Chronic restraint stress and AVE administration were applied on day 1 and lasted for 23 days. 5% minoxidil was applied on day 9 of the experiment (day 1 after depilation) and lasted for 16 days. The depilation of all mice was on day 8 of the experiment to induce anagen (The day on depilation did not apply restraint stress or drug). Behavioral test (OFT) was exmained on day 25 and tissue harvesting was collected on day 26 of the experiment

The procedure of chronic restraint stress was conducted as previously described and lasted for 23 days (Fig. 1). Mice were placed into 50 ml conical tubes without physically compressed for 6 h (10:00 a.m.-16:00 p.m.) each day [25]. During the period of stress application, control mice were kept undisturbed in their cages, and all groups of mice were not provided with food and water.

### Synchronization of hair cycle

On day 8 of the experiment, we anesthetized all mice and applied a mixture of wax and rosin (1:1 on weight) to the murine back skin (from neck to tail) to induce anagen. Then we peeled off the mixture and removed all hair shafts to induce synchronization of hair cycle [10]. Mice were not exposed to restraint stress on the day of depilation.

### Pharmaceutical intervention

Forty C57BL/6 mice were randomly assigned to four subgroups (*n* = 10 each group): (1) the non-stressed or control group (CTRL); (2) chronic restraint stress group (RS): application of chronic restraint stress without drug; (3) AVE + chronic restraint stress (AR): application of AVE (dissolved in 0.5% CMC-Na) with 80 mg/kg/day by gavage for 23 days, also applied with chronic restraint stress; (4)5% minoxidil + chronic restraint stress (MR): 100 μl of 5% minoxidil (Mandi, China) was topically applied daily on murine back skin for 16 days after depilation, also applied with chronic restraint stress. CTRL, RS and MR groups received the same volume of vehicle (0.5% CMC-Na) for 23 days in paralleled with AR group.

### Tissue collection

All animals were sacrificed by cervical dislocation under anesthesia on day 26 of the experiment (day 18 after depilation). Dorsal skin specimens were harvested about 2 × 5 cm, and then partially fixed in 4% paraformaldehyde to perform hematoxylin and eosin (H&E) staining. The remaining skin samples were immediately cryopreserved in liquid nitrogen to perform immunofluorescence and western blot analysis.

### Histological examination

Skin specimens were obtained as described above. The tissues were embedded in paraffin, and 4-μm thick sections were cut for hematoxylin and eosin staining. Then we analyzed the sections at 100× and 400× magnification by Olympus BX41 fluorescence microscope. Under representative areas at 100× magnification, hair parameters including thickness of dermis and distance between hair germ and subcutaneous layer were measured (*n* = 10/mouse). The total number of hair follicles at least 10 visual fields per mouse, 3–5 mice each group were quantified [26, 27].

### Assessment of hair cycle

The hair stage was assessed macroscopically according to their morphology of dermal papilla and sebaceous glands as described previously (anagen: anagen I = factor 1, anagen II = factor 2, anagen III = factor 3, etc.; for catagen: catagen I = factor 1, catagen II = factor 2, catagen III = factor 3, etc.) [26]. The number of hair follicles in each specific stage is multiplied by the corresponding factor. The results of each sum were totaled and divided by the total number of hair follicles counted.

### Immunofluorescence

For immunofluorescence histochemistry, serial cryosections (12 μm thick) were incubated with the primary antibodies overnight at 4 °C. Then we observed reaction products by TRITC-labeled secondary antibodies (Cwbio, Beijing, China) [26]. Cryosections were counterstained with DAPI (Invitrogen, Carlsbad, CA) for identification of cell nuclei. The following primary antibodies and concentrations were used: Ki67 antibody at 1:400 (Abcam), cytokeratin19 at 1:200 (Abcam), SP antibody at 1:10000 (Abcam), CGRP antibody at 1:400 (Abcam). PBS was used to replace the primary antibody in negative controls. Never fibers were counted by standards of structures with strong and continuous linear staining at 200× and 400× magnification by the Olympus BX41 fluorescence microscope. The number of labeled nerve fibers was counted in the dermal compartment. At least 10 visual fields per mouse, 3–5 mice each group were studied [28].

### Western blot analysis

Murine protein samples (30 μg) lysed in cold lysis buffer (1 mM phenylmethyl sulfoxide, 0.01% aprotinin, 1% Triton X-100, and 0.1 M phosphate-buffered saline, pH 6.8) were separated by 10% SDS-PAGE gel, and transferred to nitrocellulose membranes (Millipore). Then the membranes were blocked with 5% non-fat milk (containing 0.05% Tween-20) for 1 h at room temperature and incubated with corresponding primary appropriate antibodies overnight (4 °C). After washed three times with TBST (5–10 min each time), the membranes were incubated in secondary antibodies for 1 h (room temperature). Enhanced chemiluminescence detection system was used to visualize the immunoreactive bands. Quantity One (Bio-Rad) was used to quantified the protein expression from three independent experiments. The following primary antibodies were used in western blot analysis: NK-1R (1:800, Santa Cruz), TYR (1:300, Santa Cruz), TRP-1 (1:300, Abcam), TRP-2 (1:800, Abcam), MITF (1:800, Santa Cruz), and β-actin (1:1000, Sigma).

### Isolation and culture of mouse vibrissa follicles and in vitro study

Mouse vibrissae follicles were obtained from male 5-week-old C57BL/6 mice. Knife and tweezers were used to harvest the normal vibrissae hair follicles from the upper lip region. The isolated vibrissa follicles were placed for 20 min in D-Hank’s streptomycin. After washing with PBS twice, the follicles were immediately placed in 24-well microplates containing the culture medium and cultured in 5% CO_2_ for 8 days at 37 °C according to the reported method [29, 30].

The isolated vibrissae follicles were divided into 3 groups: (1) control group: only culture medium; (2) SP group: 10^− 8^ mol/L substance P + culture medium; (3) AVE group: 10 μg/ml AVE + 10^− 8^ mol/L substance P + culture medium. Each group used 10–15 follicles. The concentration of AVE and SP were chosen based on previous studies [22, 31]. The length of outgrowing hair shaft was measured daily for 8 days by analysis of digital images. In addition, western blot was used to detect the protein expression of NK-1R.

### Statistical analysis

Data were expressed as the mean ± S.E.M. Statistical significance were performed using unpaired two-tail Student’s t-test or one-way ANOVA with Tukey’s post hoc test with significance being accepted at *p* < 0.05. We used GraphPad Prism (UK) for the statistical analysis.

## Results

### Effects of AVE on hair growth in stressed C57BL/6 mice

**Fig. 2:**
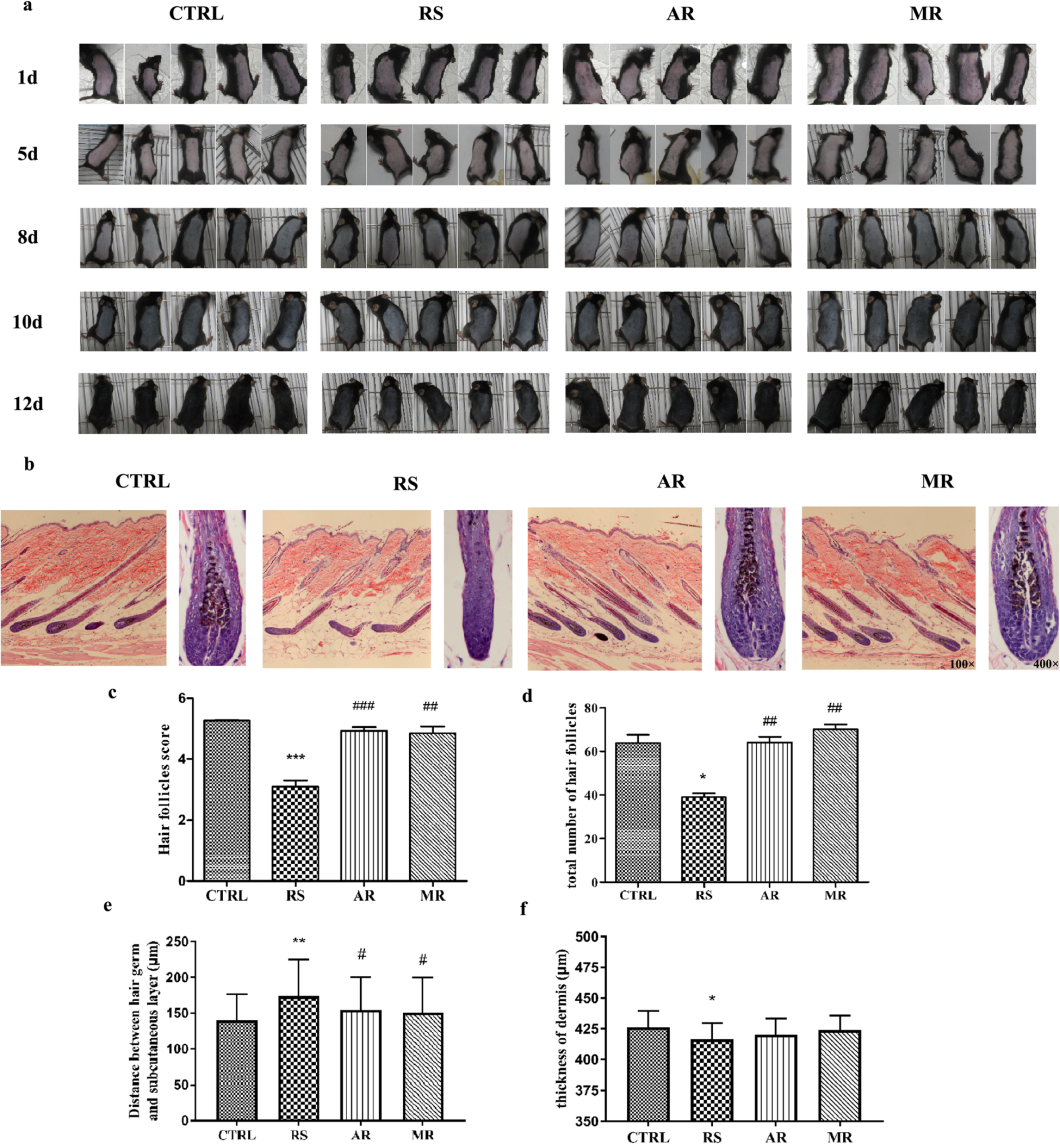
Effects of AVE on hair growth in stress-induced C57BL/6 mice. **a** Photographs showed the color change of murine dorsal skin on day 1, 5, 8, 10 and 12 after depilation. **b** H & E staining of murine dorsal skin sections showed the morphological changes in hair follicles. Magnification: 100× on the left; 400× on the right. **c** The results of hair cycle score in each group. **d** The results of total number of hair follicles in each group. **e** Distance between the hair germ and the subcutaneous layer in each group. **f** Thickness of dermis in each group. Data are presented as mean ± S.E.M, *n* = 3–5 in each group. * *p* < 0.05, **p < 0.01, ***p < 0.001 compared with CTRL group; ## *p* < 0.01, ###*p* < 0.001 compared with RS group

We took photographs daily by digital camera (Canon, Japan) to observe the murine back skin color after depilation. The murine skin color was pink in the telogen phase on day 1 after depilation, and became dark when entering into anagen. On day 18 after depilation, the hair cycle of C57BL/6 mice was during anagen-catagen transition [10]. As shown in Fig. 2a, the dorsal skin color of the CTRL group was darker than the RS group on day 12 after depilation, and the hair shafts were visible in the CTRL group. We observed murine skin color turned black in the AR and MR group on day 12 after depilation, which was similar in the CTRL group.

H & E staining of the dorsal skin sections was performed to detect the progression of hair follicles during hair cycle (Fig. 2b). According to the time-scale for hair cycle, The score of hair cycle in Fig. 2c showed that the most of hair follicles in the RS group entered into catagen II / III stage. In this stage, the dermal papilla degenerated and the hair bulb became narrower. However, most hair follicles of the CTRL, AR and MR groups were still in anagen VI, indicating that AVE treatment counteract the stress-induced hair follicle inhibition. CRS significantly decreased hair cycle score in the RS group (*p* < 0.001). Both AVE and 5% minoxidil counteracted this effect caused by CRS (*p* < 0.001, *p* < 0.01). As shown in Fig. 2d, CRS significantly decreased the total number of murine hair follicles compared with the CTRL group (*p* < 0.05). Besides, the total number of hair follicles in the AR and MR groups were increased compared with the RS group (*p* < 0.01). Statistical analysis of Fig. 2e demonstrated that CRS increase the length of hair germ and subcutaneous layers significantly (*p* < 0.01), whereas this hair parameter was obviously decreased in the AR and MR group compared with the RS group. Figure 2f showed no significance in thickness of dermis among four groups (*p* > 0.05).

The above results indicate that AVE can promote hair follicle growth by inducing the conversion of telogen to anagen and inhibiting catagen premature.

### Effects of AVE on Ki67^+^ keratinocytes and cytokeratin19^+^ stem cells proliferation in stressed C57BL/6 mice

**Fig. 3:**
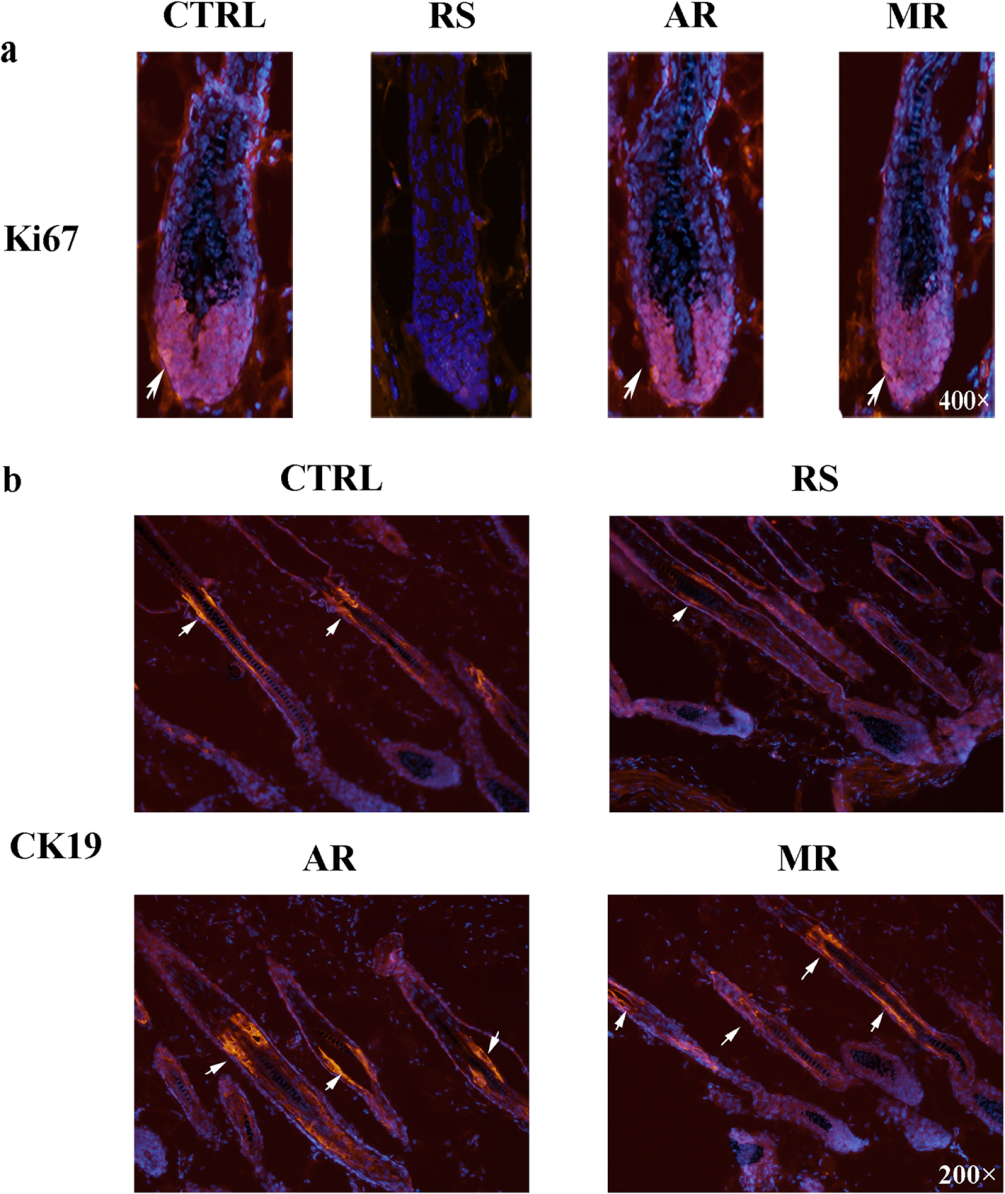
Effects of AVE on Ki67^+^ kertinocytes and cytokeratin19 (CK19)^+^ stem cells proliferation. The expressions of Ki67^+^ kertinocytes (red staining, pointed out by arrows) (**a**) and CK19^+^ stem cells (red staining, pointed out by arrows) (**b**) in the hair follicles of C57BL/6 mice were visualized by immunofluorescence. Magnification: (**a**) 400×; (**b**) 200×. Negative controls did not reveal specific immunoreactivity

The immunofluorescence of Ki67^+^ keratinocytes indicated the situation of cell proliferation in hair bulb region. Compared to the RS group, there are high numbers of Ki67^+^ keratinocytes in the CTRL group (Fig. 3a). Both AVE and 5%minoxidil increased the number of Ki67^+^ keratinocytes in hair bulb region. In addition, we visualized the stem cells located in the hair bulge areas, marked by cytokeratin19 (CK19) positivity. CRS decreased the positive cells in bulge areas compared to the CTRL group (Fig. 3b). The expression and location of CK19^+^ cells in the AR and MR group were similar to the CTRL group. These evidences indicated that the application of AVE can promote hair follicle growth via Ki67^+^ keratinocytes and CK19^+^ stem cells proliferation.

### Effects of AVE on melanogenesis in stress-induced C57BL/6 mice

Melanocytes only locate in the bulb region of hair follicles in C57BL/6 mice, and melanin synthesis is coupled to anagen, ceases during catagen, and is absent during telogen during the hair cycle [32]. To investigative the effects of AVE on melanogenesis in murine hair follicles, western blot analysis was performed to detect the expressions of TYR, TRP-1, TRP-2 and MITF, which are the key elements in melanogenesis. The seconded (lower one) band of TYR was the specified one, and the first one (upper one) may be the glycosylated tyrosinase band due to the primary antibody was an affinity purified goat polyclonal antibody. We found expression levels of TYR, TRP-1, TRP-2 and MITF proteins in murine hair follicles were significantly decreased after exposure of CRS compared with the CTRL group (*p* < 0.05). After AVE and 5% minoxidil treatment, expressions of TYR, TRP-1, TRP-2 and MITF proteins were significantly increased compared with the RS group (*p* < 0.05, *p* < 0.01). Compared with the RS group, a higher level of significance was observed in the expressions of TYR and TRP-2 by application of AVE (*p* < 0.01). These results suggest that AVE counteract the stress-induced melanogenesis inhibition via up-regulating the expressions of melanogenic proteins.

### Effects of AVE on peptidergic innervation in mice dorsal skin

**Fig. 4:**
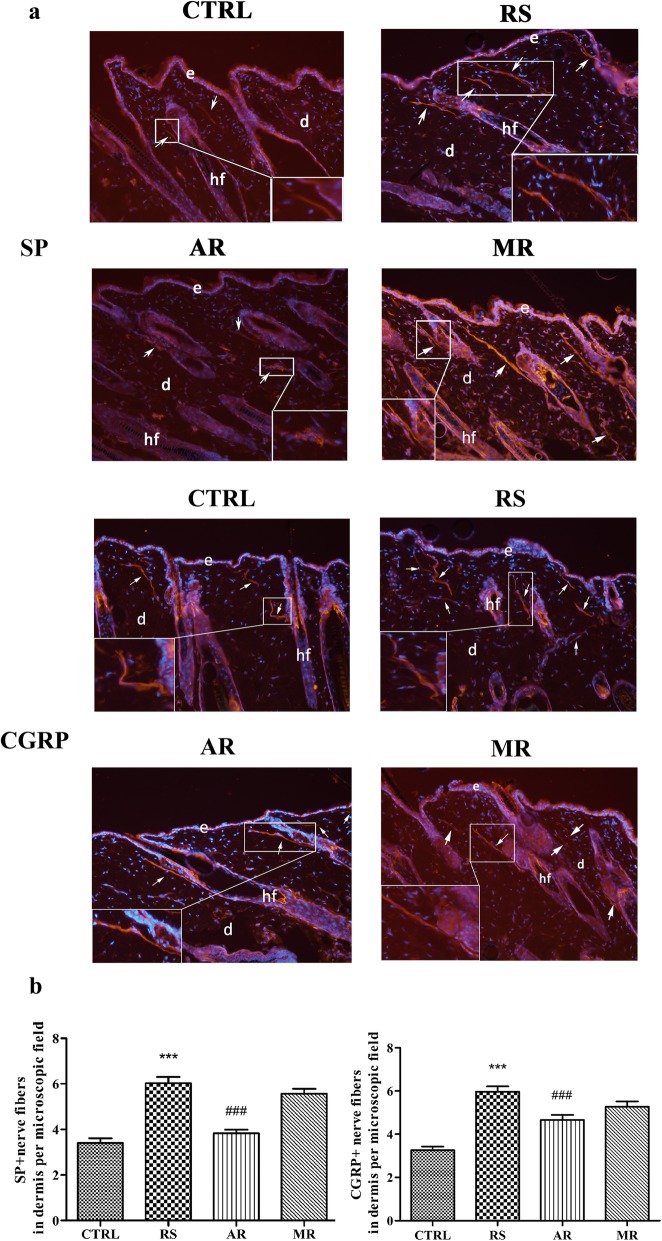
Effects of AVE on melanogenic proteins in murine dorsal skin. **a** Representative western blot analysis of the expressions of TYR, TRP-1, TRP-2 and MITF. **b** Quantitation of western blot analysis for TYR, TRP-1, TRP-2 and MITF. Equal protein loadings were confirmed using β-actin antibody. Total skin proteins (30 μg) were subjected to 10% SDS-PAGE. Densitometric scanning of band intensities obtained from three separate experiments by the Quantity One 1D analysis software program was used to quantify the change of proteins expressions (control value taken as onefold in each case). The data showed the mean ± S.E.M of three experiments. * *p* < 0.05 compared with CTRL group; # *p* < 0.05, ## *p* < 0.01 compared with RS group

To investigative the possible neurobiological mechanism of AVE in promoting hair follicle growth, we examined the expressions of SP^+^ and CGRP^+^ nerve fibers in murine back skin. SP^+^ and CGRP^+^ nerve fibers were detected in the dermis of murine skin (Fig. 4a). Compared with the CTRL group, the SP^+^ nerve fibers number of the RS group was significantly increased (*p* < 0.001) (Fig. 4b). AVE administration significantly decreased the SP^+^ nerve fibers number compared with the RS group (*p* < 0.001). Whereas, the reduced number of SP^+^ nerve fibers did not reach a level of statistical significance between the MR and RS group (*p* > 0.05). Similar results were observed in CGRP^+^ nerve fibers among four groups. This phenomenon suggested that CRS cause the increase of peripheral nerve SP and CGRP expressions, and application of AVE counteracted these effects.

### AVE counteracted the inhibited effects of hair follicle growth caused by substance P in vitro

**Fig. 5:**
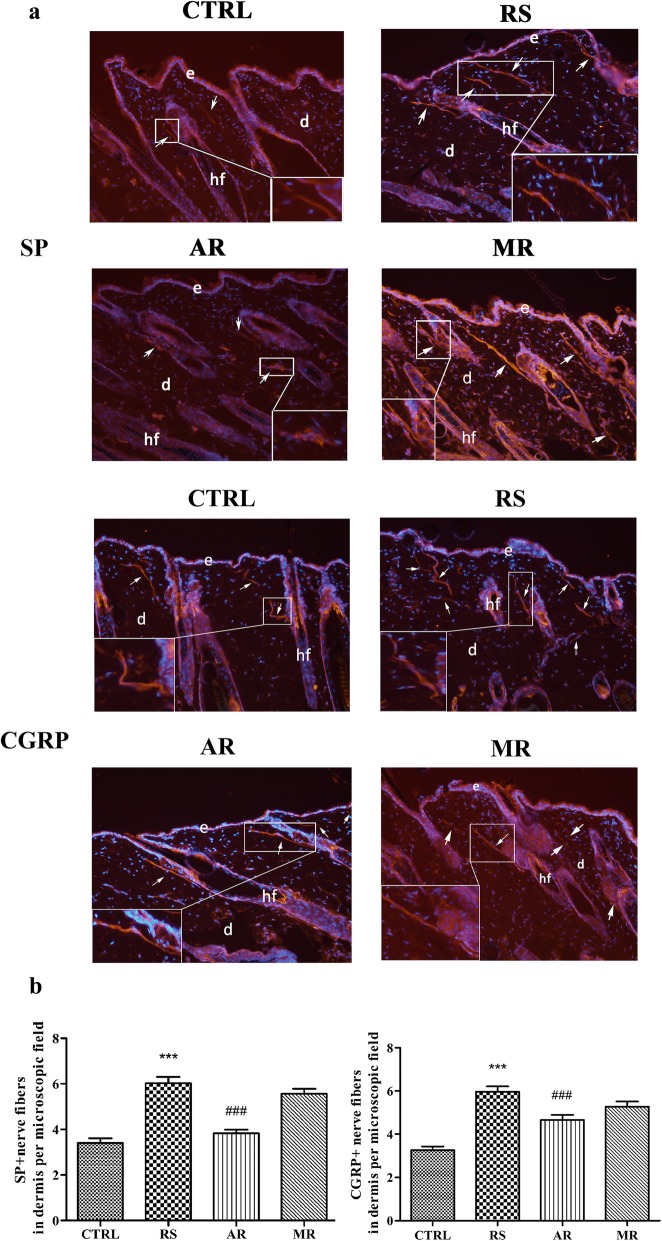
Effects of AVE on peptidergic innervation in murine dorsal skin. Histochemical detection reveals the location and expressions of SP^+^ and CGRP^+^ nerve fibers (red staining, pointed out by arrows) by immunofluorescence (**a**), and the number of SP^+^ and CGRP^+^ nerve fibers per visual field in the skin from four mice groups were measured (**b**). In the immunofluorescence micrographs, nerve fibers are labeled in red, and cell nuclei are labeled blue with DAPI. The number of nerve fibers was quantified at least 10 visual fields per mouse, 3–5 mice each group. Results shown are mean ± S.E.M. ***p < 0.001 compared with control; ###*p* < 0.001 compared with RS group. Abbreviations: e, epidermis; d, dermis; hf, hair follicle. Magnification: 200 ×

In previous study, Peters et al. confirmed that treatment with 10^− 8^ and 10^− 10^ mol/L SP can cause a significant reduced hair shaft elongation and a dose-dependent inhibition [31]. Thus, we selected SP with 10^− 8^ mol/L as a catagen-inducing factor to cause inhibited effects in the hair organ culture. On day 8 of the culture, we found 10^− 8^ mol/L SP significantly inhibited hair growth compared with the control group (*p* < 0.05) (Fig. 5a), whereas 10 μg/ml AVE counteracted the reduction of hair shaft elongation caused by substance P (*p* < 0.05). To further explorer the mechanism, we examined the protein expression of cognate receptor NK-1R in cultured hair follicles. Our result shown that compared with the SP group, application of AVE down-regulated the expression of NK-1R protein (*p* < 0.01) (Fig. 5b). These results indicate that AVE also counteract murine hair follicle growth inhibition caused by substance P*in vitro*.

## Discussion

Recently, studies on natural products have been accumulating because of their less side effects and increased safety [33]. Previous studies have confirmed the alcohol extract of *Vernonia anthelmintica (L.) willd seed* (AVE) has hair growth promoting activity [23]. However, whether AVE could have effects on stress-induced hair follicle growth inhibition has not been studied, and its molecular mechanism is still unknown. In this study, we show for the first time that AVE, a plant-based pharmaceutical product, promotes hair follicle growth in chronic restraint stress-induced C57BL/6 mice in vivo. Furthermore, AVE can counteract the hair growth inhibition caused by substance P in an in vitro analysis.

As a highly sensitive mini-organ, the hair follicle undergoes continuous cycles. This process consists of three stages, including phases of rapid growth (anagen), apoptosis-driven regression (catagen) and relative quiescence (telogen). Lots of research have reported that hormones, neuropeptides and cytokines may influence the hair follicle growth and hair cycle [9]. To investigate the effect of AVE on hair follicle growth inhibition after the exposure of psychological stress, a chronic restraint stressed mice model was employed to induce emotional effects on mice with no harm to biological injury [34]. In an in vivo study, 5% minoxidil was used as a positive drug to compare its effects with AVE. Minoxidil is an adenosine triphosphate sensitive potassium channel opener, and is one of the only two drugs for alopecia treatment approved by Food and Drug Administration [35]. It has been confirmed that minoxidil can increase the blood flow of balding scalps, prolong the anagen stage and enhance hair growth by activating Erk and Akt signaling which enhance the survival of cultured dermal papilla cells, and increase in the Bcl-2/Bax ratio protecting cells against cell death [36–38]. However, no literature has reported its effect or mechanism of action on central or peripheral nervous system. Considering the possible systematic neurobiological mechanism and the duration of treatment of AVE had been studied previously, we chose intragastric administration for 23 days. In addition, we applied minoxidil after depilation for the reason of it is a widely used as a tropical agent.

We proved that both administration of AVE and 5%minoxidil can alter hair cycle and promote hair follicle growth in C57BL/6 mice after the exposure of CRS (Fig. 2). Most hair follicles of the RS group entered into catagen II/III on day 18 after depilation (Fig. 2b and c). In addition, CRS significantly decreased the total number of hair follicles (Fig. 2d), and increase the length of hair germ and subcutaneous layers (Fig. 2e and f). AVE counteracted this stress-induced hair follicle inhibition, and most of them stayed in the stage of anagen VI. In addition, we found that CRS down-regulate the Ki67^+^ keratinocytes number in the hair bulb (Fig. 3a) and CK19^+^ stem cells number in the hair bulge (Fig. 3b) of hair follicles by immunofluorescence. After administration of AVE and 5% minoxidil, increasing number of Ki67^+^ keratinocytes and CK19^+^ stem cells were observed in stressed mice. As an important indicator for the homeostasis of self-renewing tissues, hair follicle stem cells can be detected in hair bulge and marked by CK19 positivity [39]. During hair follicle growth and regeneration, these cells play an important role in forming the epidermal, follicle, and sebaceous gland [40]. Thus, the promoting effects of Ki67^+^ keratinocytes and CK19^+^ stem cells proliferation of AVE may contribute to the hair regrowth.

**Fig. 6:**
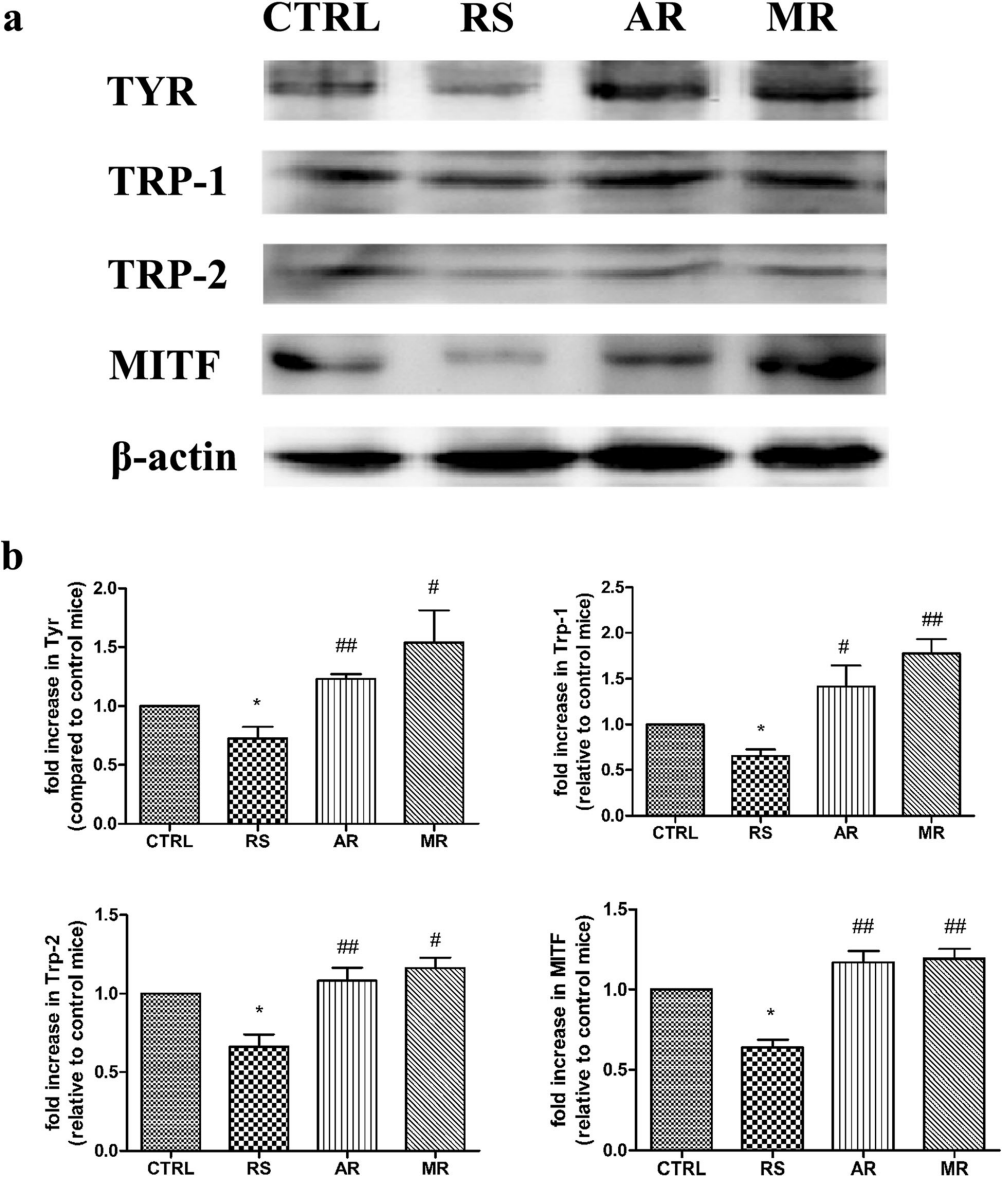
AVE counteracted the inhibited effect of hair follicle growth caused by substance P in vitro. Mouse vibrissa follicles from 5-week-old male C57BL/6 mice were isolated and cultured. Each group used 10–15 follicles. **a** The length of vibrissa follicles was measured daily for 8 days. ●, the control group; ▲, addition of 10^− 8^ mol/L SP; ■, addition of 10^− 8^ mol/L SP and 10 μg/ml AVE. *p < 0.05 compared with control group; #*p* < 0.05 compared with SP group. **b** Western blot analysis was used to examine the expression of NK-1R in murine vibrissa follicles. Equal protein loadings were confirmed using β-actin antibody. Densitometric scanning of band intensities obtained from three separate experiments by the Quantity One 1D analysis software program was used to quantify the change of proteins expressions (control value taken as onefold in each case). The data showed the mean ± S.E.M of three experiments. *p < 0.05 compared with control group; ##*p* < 0.01 compared with SP group

Other from human, all the melanocytes derived from hair follicles in C57BL/6 mice [41]. In murine hair follicles, melanin synthesis is coupled to the anagen, ceases during catagen, and is absent throughout telogen. Therefore, murine skin color appears to be pink in telogen and changes to be black in the anagen stage [32, 42]. Tyrosinase has been known as the key enzyme during the process for the synthesis of melanin pigment [43]. Furthermore, the tyrosinase family genes TYR, TRP-1, TRP-2 responsible for pigmentation are transcriptionally regulated by microphthalmia-associated transcription factor (MITF) [44, 45]. In our study, both AVE and 5% minoxidil treatment promoted melanogenesis in murine hair follicles by significantly increased the expressions of melanogenic proteins (Fig. 6). This finding implied that more hair follicles stay in anagen stage of AR group compared with the RS group, and AVE can counteract the catagen premature. This result was consistent with our previous study about the effects of AVE on hair follicle growth.

After confirmed the direct hair follicle-promoting effects of AVE, we further investigated its possible neurobiological mechanism. The central nervous stress perception is translated to skin not only via classical stress hormones but also via neuropeptides [46]. Recent studies have proved that remodeling of hair follicle innervations contribute to hair follicle cycling, and peptidergic innervations are located around the bulge region [15, 28, 47]. Here we identified distinct sensory neuropeptide distribution in murine dorsal skin (Fig. 4a). In our results, CRS significantly increased the numbers of SP^+^ and CGRP^+^ nerve fibers. Application of AVE significantly reduced the number of these nerve fibers, whereas 5% minoxidil had no such effect (Fig. 4b). Thus we suggest that AVE may affect hair follicle growth and hair cycle via the modulation of neuropeptides, which is different from the pathway affected by minoxidil.

Murine hair follicle growth can be manipulated by neuropepitide as substance P both in vivo and in vitro [48, 49]*.* Substance P (SP) is not only a neuropeptide with pain-mediating function, but also play an important role in various stress-related disorders [50]. Literatures have reported that SP inhibit hair matrix kertinocytes and hair follicles express NK1 receptors [51]. Furthermore, SP may i indirectly cause human hair growth inhibition through up-regulating p75NTR and NGF and down-regulating TrkA expressions [52–54]. Therefore, we used SP as a mediator in murine vibrissa follicles culture to inhibit hair growth. In our organ culture model, we found AVE can counteract the inhibited effect of SP through promoting hair shaft growth and down-regulating the protein expression of NK-1R.

Up to date, clinical and experimental observations provide evidence for complex molecular mechanisms involved in the pathogenesis of hair loss after stress stimuli. Traditional tropical treatment of drugs for hair disorders may not reach perfect curative effect. Thus, exploring novel pathways may offer us more strategies in stress-triggered alopecia treatment and to discover new candidates. In our study, AVE counteracted stress-induced murine hair follicle growth inhibition via inducing the conversion of telogen to anagen and inhibiting catagen premature, increasing bulb keratinocytes and bulge stem cells proliferation, promoting melanogenesis, and reducing SP^+^ and CGRP^+^ nerve fibers. Furthermore, AVE also counteracted murine hair follicle growth inhibition caused by substance P in vitro. Therefore, AVE may be an effective new candidate for clinical treatment of human alopecia caused by psychoemotional stress. Further works will be necessary to investigate the function of individual active components of AVE and to identify their mechanism of action.

## Conclusions

These results suggest that AVE counteract stress-induced hair follicle growth inhibition in C57BL/6 mice in vivo and in vitro, and may be an effective new candidate for treatment of human alopecia caused by stress.

## Supplementary information

**Additional file 1: Figure S1.** Effect of AVE on mice body weight. Mice body weight was measured on day 1, 5, 9, 13, 17, 21 and 25 of the experiment.

**Additional file 2: Figure S2.** Effects of AVE on immune index in stressed C57BL/6 mice. (a) Effects of AVE on spleen index in stressed C57BL/6 mice; (b) Effects of AVE on thymus index in stressed C57BL/6 mice. *p < 0.05 compared with CTRL group, #*p* < 0.01 compared with RS group.

## Data Availability

The datasets used and/or analyzed during the current study are available from the corresponding author upon reasonable request.

## Abbreviations

Akt: Protein kinase B
AR: AVE+ restraint stress
AVE: Alcohol extract of *Vernonia anthelmintica* (L.) willd
Bax: Bcl-2 associated X protein
Bcl-2: B-cell lymphoma-*2*
CGRP: Calcitonin gene-related peptide
CK19: Cytokeratin19
CMC-Na: Sodium carboxyl methyl cellulose
CTRL: Control
DAPI: 4′,6-diamidino-2-phenylindole
Erk: Extracellular signal-regulated kinase;
FDA: Food and Drug Administration;
H&E: Hematoxylin-eosin
HPLC: High performance liquid chromatography;
Ki67: Nuclear-associated antigen;
MITF: Microphthalmia-associated transcription factor
MR: 5%minoxidil+ restraint stress
NGF: Nerve growth factor
NK-1R: Neurokinin-1 receptor
OFT: Open-field test
p75NTR: p75 neurotrophin receptor
PBS: Phosphate buffered solution
RS: Restraint stress
S.E.M.: Standard error of mean
SDS: Sodium dodecyl sulfate
SP: Substance P
TRITC: Tetramethylrhodamine
TrkA: Tyrosine kinase
TRP-1: Tyrosinase-related protein-1
TRP-2: Tyrosinase-related protein-2
TYR: Tyrosinase
