# Supplementary figures and images for: Alcohol extract from Vernonia anthelmintica willd (L.) seed counteracts stress-induced murine hair follicle growth inhibition
Source: BMC Complement Altern Med. 2019 Dec 17;19:372. doi: 10.1186/s12906-019-2744-9 (PMC6918677; doi:10.1186/s12906-019-2744-9)

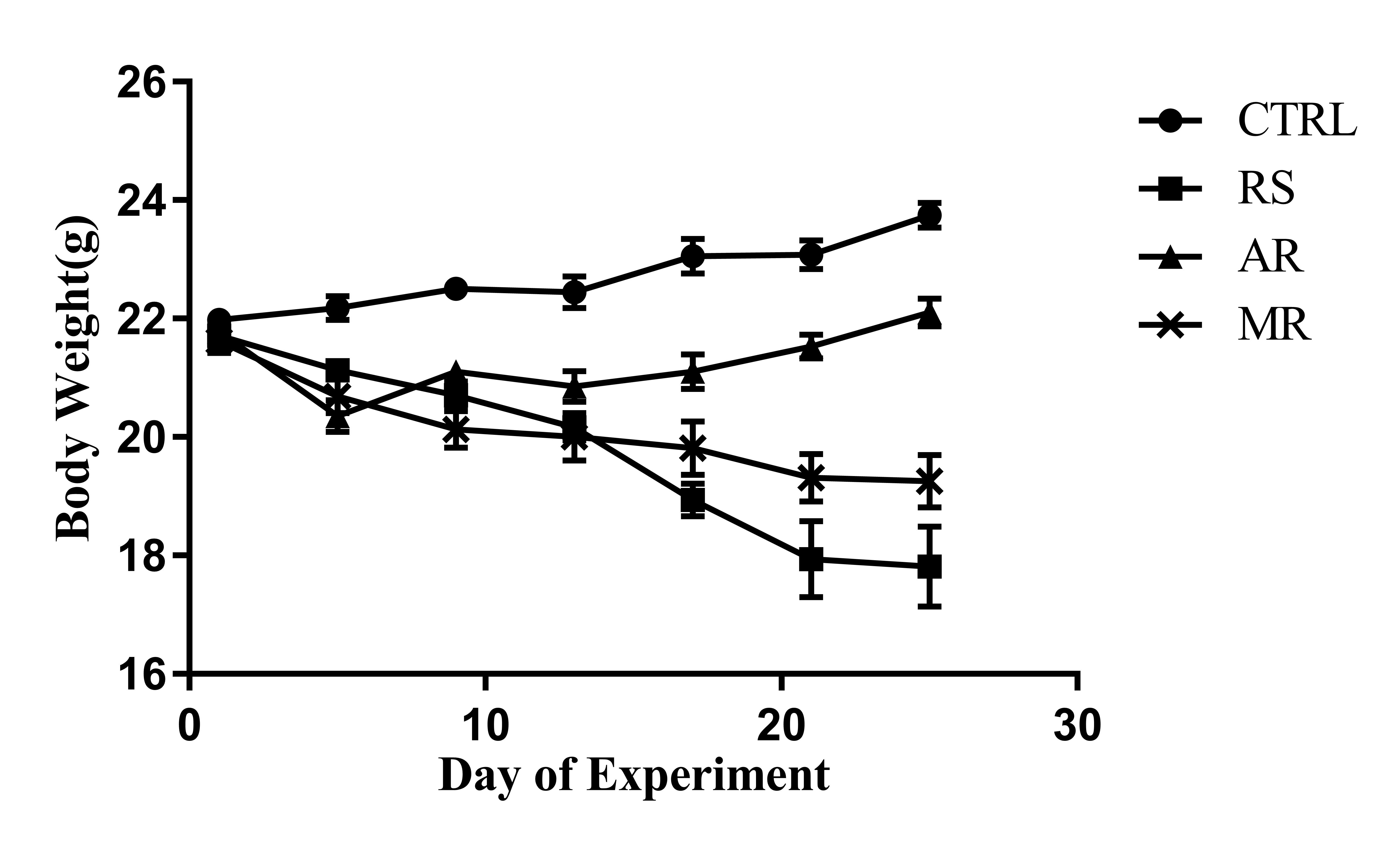

Supplement: Supplementary file 1 — Additional file 1: Figure S1. Effect of AVE on mice body weight. Mice body weight was measured on day 1, 5, 9, 13, 17, 21 and 25 of the experiment. [file 12906_2019_2744_MOESM1_ESM.jpg]

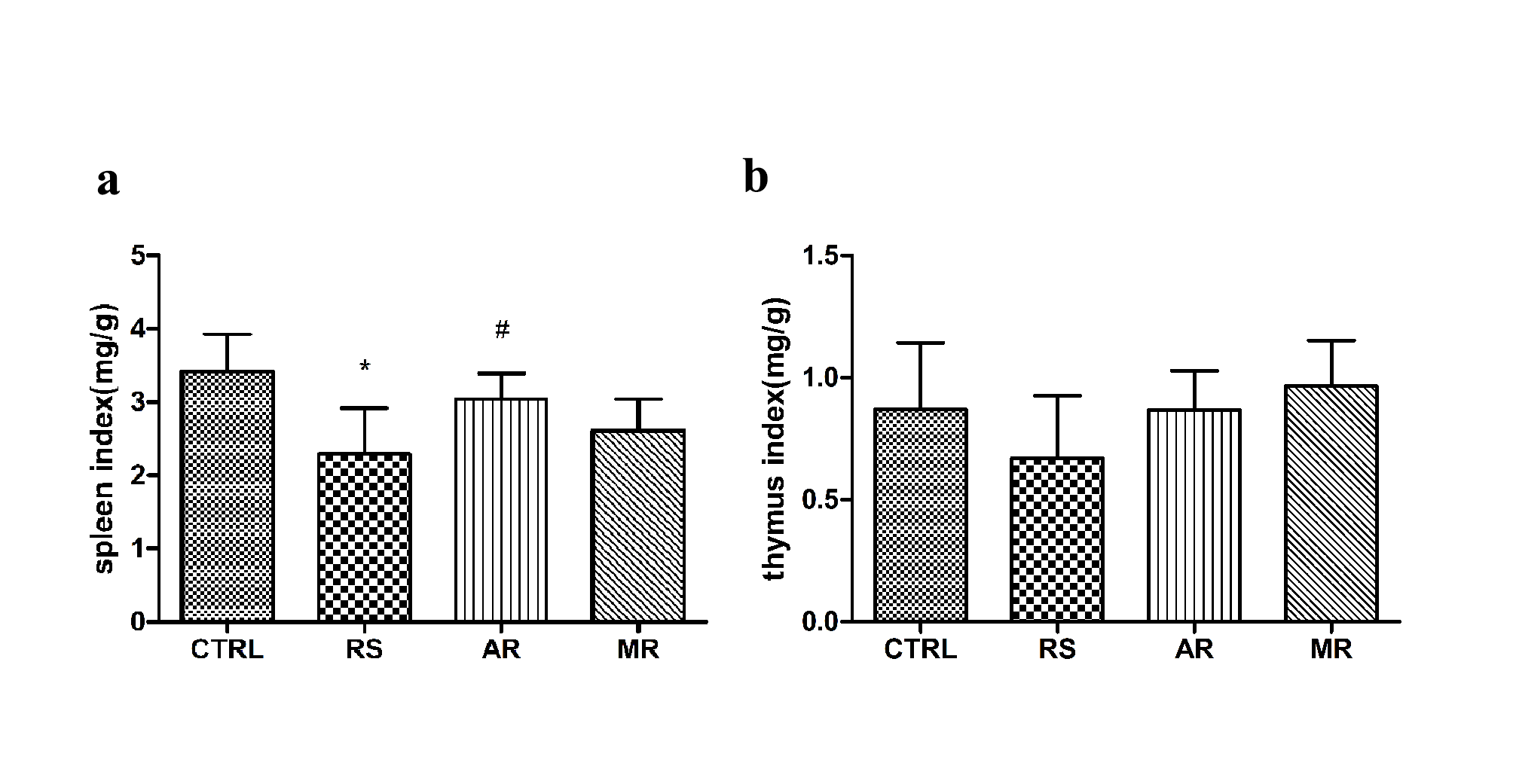

Supplement: Supplementary file 2 — Additional file 2: Figure S2. Effects of AVE on immune index in stressed C57BL/6 mice. (a) Effects of AVE on spleen index in stressed C57BL/6 mice; (b) Effects of AVE on thymus index in stressed C57BL/6 mice. *p < 0.05 compared with CTRL group, #p < 0.01 compared with RS group. [file 12906_2019_2744_MOESM2_ESM.tif]
